# The genome sequence of an orb-weaver spider, *Araneus angulatus* Clerck, 1757 (Araneae: Araneidae)

**DOI:** 10.12688/wellcomeopenres.25196.1

**Published:** 2025-11-21

**Authors:** Christopher M. Raper, Olga Sivell, Natural History Museum Genome Acquisition Lab, Darwin Tree of Life Barcoding Collective, Wellcome Sanger Institute Tree of Life Management, Samples and Laboratory team, Wellcome Sanger Institute Scientific Operations: Sequencing Operations, Wellcome Sanger Institute Tree of Life Core Informatics team, Tree of Life Core Informatics collective, Darwin Tree of Life Consortium

**Affiliations:** 1 Natural History Museum, London, England, UK

**Keywords:** Araneus angulatus; orb-weaver spider; genome sequence; chromosomal; Araneae

## Abstract

We present a genome assembly from an individual female *Araneus angulatus* (orb-weaver spider; Arthropoda; Arachnida; Araneae; Araneidae). The assembly contains two haplotypes with total lengths of 2 980.91 megabases and 2 941.08 megabases. Most of haplotype 1 (94.74%) is scaffolded into 28 chromosomal pseudomolecules. Haplotype 2 was assembled to scaffold level. The mitochondrial genome has also been assembled, with a length of 14.53 kilobases. This assembly was generated as part of the Darwin Tree of Life project, which produces reference genomes for eukaryotic species found in Britain and Ireland.

## Species taxonomy

Eukaryota; Opisthokonta; Metazoa; Eumetazoa; Bilateria; Protostomia; Ecdysozoa; Panarthropoda; Arthropoda; Chelicerata; Arachnida; Araneae; Araneomorphae; Entelegynae; Orbiculariae; Araneoidea; Araneidae; Araneus; Araneus angulatus Clerck, 1757 (NCBI:txid1112382)

## Background

*Araneus angulatus* Clerck 1757 is a species from family Araneidae (orbweb spiders or orb-weavers) and a type species of the genus *Araneus* Clerck, 1757. This is a fairly large spider, with a body length of 12–19 mm in females, and 10–12 mm in males ( Roberts, 1996). The spider is greyish-brown in colour, with reddish-brown carapace covered with long white hairs. The pale brown abdomen has a pair of distinct raised “humps”, somewhat similar to *Gibbranea gibbosa*, and a pattern tapering posteriorly, occasionally with white markings medially ( Bee *et al.*, 2017; Bee *et al.*, 2020). Males are similar in appearance to females, however with a smaller abdomen and generally darker in colour. The structure of the epigyne and male palpal organ are distinctive and pictured in Roberts (1996).

*Araneus angulatus* occurs in Europe, North Africa, Turkey, Russia (Europe to Far East), Iran, Central Asia and Korea ( Nentwig *et al.*, 2025a; World Spider Catalogue, 2025). In Britain it is very local and scarce, mostly restricted to southern coastal counties in England, also recorded from south Wales and Surrey. It occurs in deciduous woodland, occasionally on pine and scrub on maritime heath ( Bee *et al.*, 2017; Bee *et al.*, 2020; British Arachnological Society, 2025; Dawson *et al.*,). The webs can be built high up in the trees, suspended from a long frame thread ( Bee *et al.*, 2017; Bee *et al.*, 2020).

This species is considered Nationally Scarce in Britain ( British Arachnological Society, 2025; Harvey *et al.*, 2017), and is included on the Red List for Sweden ( Gärdenfors, 2000). In Britain the males have been recorded from July to August, females from March to August; both sexes mature in June, and mature females can be encountered till September ( Bee *et al.*, 2017; Bee *et al.*, 2020; British Arachnological Society, 2025).

According to the World Spider Catalogue (2025), there are currently 198 genera recognised within Araneidae and the genus *Araneus* includes 550 species. The family Araneidae and the genus *Araneus* are both paraphyletic according to Scharff *et al*. (2020); Scharff & Coddington, (1997), while Dimitrov *et al*. (2017) and Wheeler *et al*. (2017) consider the family to be monophyletic. The phylogenetic relationships and classification of the araneids require more extensive and in-depth research.

The recent phylogenetic analysis of the Araneidae ( Scharff *et al.*, 2020) did not include *A. angulatus* due to the lack of suitable samples. The sequences published more recently on BOLD and GenBank suggest the *A. angulatus* may be more than one species ( Ratnasingham *et al.*, 2024; Scharff *et al.*, 2020). However, it is also possible that some of the specimens of other species, e.g. *A. pallidus*, might have been misidentified as *A. angulatus*. *Araneus angulatus pallidus* (Franganillo, 1909), *A. a. crucinceptus* (Franganillo, 1909), *A. a. fuscus* (Franganillo, 1909), *A. a. iberoi* (Franganillo, 1909), *A. a. castaneus* (Franganillo, 1909), previously considered subspecies of *A. angulatus*, have since been synonymised with *Araneus pallidus* (Olivier, 1789), a variable species with several colour forms, occurring in France, Portugal, Spain, Algeria and Morocco ( Breitling *et al.*, 2016; Nentwig *et al.*, 2025b; World Spider Catalogue, 2025; Wunderlich, 2023). *Araneus angulatus afolius* (Franganillo, 1909), *A. a. levifolius* (Franganillo, 1909), *A. a. niger* (Franganillo, 1918) and *A. a. nitidifolius* (Franganillo, 1909) were synonymised with *A. angulatus* ( Breitling *et al.*, 2016). Characters distinguishing *A. angulatus* and *A. pallidus* as well as different colour forms are discussed by Breitling *et al*. (2016), the pedipalpus of *A. pallidus* is figured in Grasshoff (1968) and Wunderlich (2023) and a key to *Araneus* Clerck, 1757 is available online.

The identity of *A. angulatus* is important for our understanding of the phylogeny and taxonomy of Araneidae and this publication aims to contribute to the body of knowledge by providing a high-quality genome of *A. angulatus* from Britain. The mitochondrial genome of this species was sequenced from a specimen collected from China ( Wang *et al.*, 2019).

**Figure 1.**
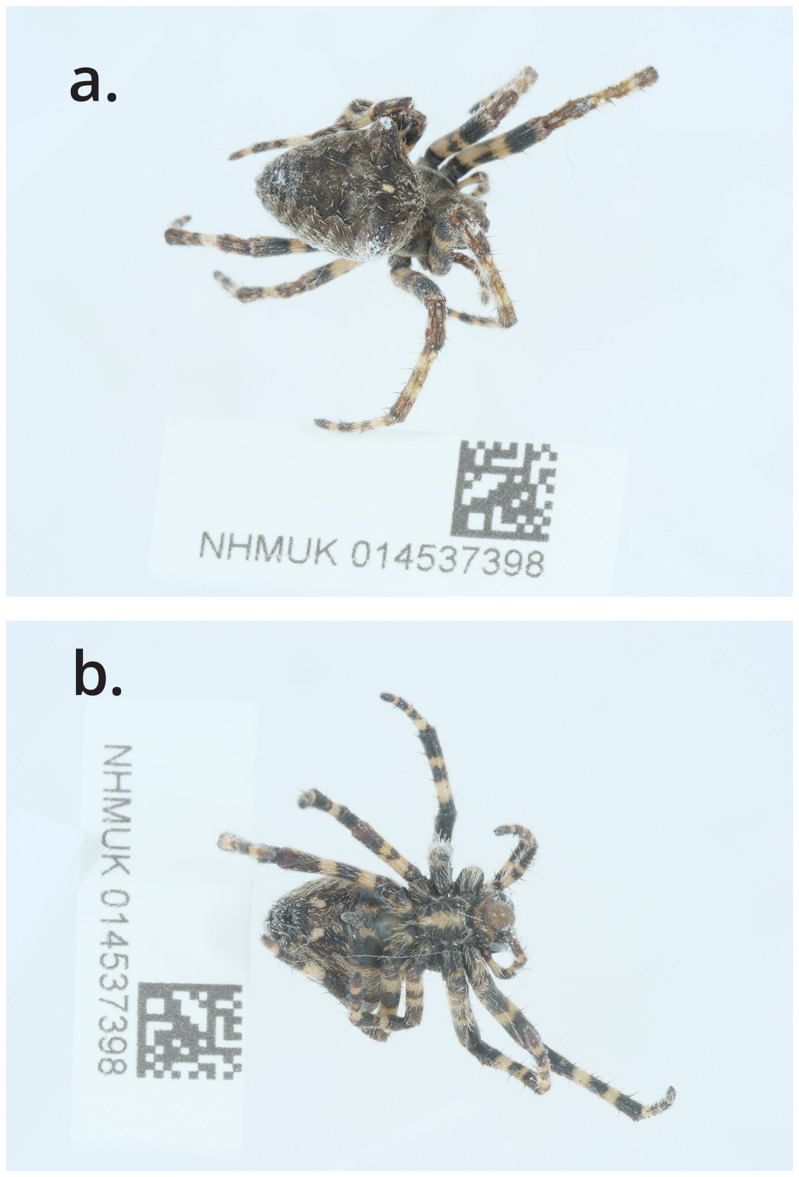
Photographs of the *Araneus angulatus* (qqAraAngu1) specimen used for genome sequencing.

The high-quality genome of *Araneus angulatus* was sequenced from a single male (NHMUK014537398; SAMEA11024980) from Kings Wood, St Austell, England ( Figure 1). The genome of *A. angulatus* presented here was sequenced as part of the Darwin Tree of Life Project, a collaborative effort to sequence all named eukaryotic species in the Atlantic Archipelago of Britain and Ireland. It will aid research into phylogeny of spiders and taxonomy, biology and ecology of the species.

## Methods

### Sample acquisition and DNA barcoding

The specimen used for genome sequencing was an adult female *Araneus angulatus* (specimen ID NHMUK014537398, ToLID qqAraAngu1; Figure 1), collected from Kings Wood, St Austell, England, United Kingdom (latitude 50.3045, longitude –4.8001) on 2021-06-28. The specimen was collected and identified by Chris Raper and Chris Spilling, and processed at the Natural History Museum before being shipped to the Wellcome Sanger Institute. Sample metadata were collected in line with the Darwin Tree of Life project standards described by Lawniczak *et al*. (2022).

The initial identification was verified by an additional DNA barcoding process according to the framework developed by Twyford *et al.* (2024). A small sample was dissected from the specimen and stored in ethanol, while the remaining parts were shipped on dry ice to the Wellcome Sanger Institute (WSI) (see the protocol). The tissue was lysed, the COI marker region was amplified by PCR, and amplicons were sequenced and compared to the BOLD database, confirming the species identification ( Crowley *et al.*, 2023). Following whole genome sequence generation, the relevant DNA barcode region was also used alongside the initial barcoding data for sample tracking at the WSI ( Twyford *et al.*, 2024). The standard operating procedures for Darwin Tree of Life barcoding are available on protocols.io.

### Nucleic acid extraction

Protocols for high molecular weight (HMW) DNA extraction developed at the Wellcome Sanger Institute (WSI) Tree of Life Core Laboratory are available on protocols.io ( Howard *et al.*, 2025). The qqAraAngu1 sample was weighed and triaged to determine the appropriate extraction protocol. Tissue from the abdomen was homogenised by powermashing using a PowerMasher II tissue disruptor. HMW DNA was extracted in the WSI Scientific Operations core using the Automated MagAttract v2 protocol. DNA was sheared into an average fragment size of 12–20 kb following the Megaruptor®3 for LI PacBio protocol. Sheared DNA was purified by automated SPRI (solid-phase reversible immobilisation). The concentration of the sheared and purified DNA was assessed using a Nanodrop spectrophotometer and Qubit Fluorometer using the Qubit dsDNA High Sensitivity Assay kit. Fragment size distribution was evaluated by running the sample on the FemtoPulse system. For this sample, the final post-shearing DNA had a Qubit concentration of 70.8 ng/μL and a yield of 3 405.48 ng, with a fragment size of 12.5 kb.

### PacBio HiFi library preparation and sequencing

Library preparation and sequencing were performed at the WSI Scientific Operations core. Libraries were prepared using the SMRTbell Prep Kit 3.0 (Pacific Biosciences, California, USA), following the manufacturer’s instructions. The kit includes reagents for end repair/A-tailing, adapter ligation, post-ligation SMRTbell bead clean-up, and nuclease treatment. Size selection and clean-up were performed using diluted AMPure PB beads (Pacific Biosciences). DNA concentration was quantified using a Qubit Fluorometer v4.0 (ThermoFisher Scientific) and the Qubit 1X dsDNA HS assay kit. Final library fragment size was assessed with the Agilent Femto Pulse Automated Pulsed Field CE Instrument (Agilent Technologies) using the gDNA 55 kb BAC analysis kit.

The sample was sequenced using the Sequel IIe system (Pacific Biosciences, California, USA). The concentration of the library loaded onto the Sequel IIe was in the range 40–135 pM. The SMRT link software, a PacBio web-based end-to-end workflow manager, was used to set-up and monitor the run, and to perform primary and secondary analysis of the data upon completion.

### Hi-C

***Sample preparation and crosslinking***

The Hi-C sample was prepared from 20–50 mg of frozen cephalothorax tissue of the qqAraAngu1 sample using the Arima-HiC v2 kit (Arima Genomics). Following the manufacturer’s instructions, tissue was fixed and DNA crosslinked using TC buffer to a final formaldehyde concentration of 2%. The tissue was homogenised using the Diagnocine Power Masher-II. Crosslinked DNA was digested with a restriction enzyme master mix, biotinylated, and ligated. Clean-up was performed with SPRISelect beads before library preparation. DNA concentration was measured with the Qubit Fluorometer (Thermo Fisher Scientific) and Qubit HS Assay Kit. The biotinylation percentage was estimated using the Arima-HiC v2 QC beads.

***Hi-C library preparation and sequencing***

Biotinylated DNA constructs were fragmented using a Covaris E220 sonicator and size selected to 400–600 bp using SPRISelect beads. DNA was enriched with Arima-HiC v2 kit Enrichment beads. End repair, A-tailing, and adapter ligation were carried out with the NEBNext Ultra II DNA Library Prep Kit (New England Biolabs), following a modified protocol where library preparation occurs while DNA remains bound to the Enrichment beads. Library amplification was performed using KAPA HiFi HotStart mix and a custom Unique Dual Index (UDI) barcode set (Integrated DNA Technologies). Depending on sample concentration and biotinylation percentage determined at the crosslinking stage, libraries were amplified with 10–16 PCR cycles. Post-PCR clean-up was performed with SPRISelect beads. Libraries were quantified using the AccuClear Ultra High Sensitivity dsDNA Standards Assay Kit (Biotium) and a FLUOstar Omega plate reader (BMG Labtech).

Prior to sequencing, libraries were normalised to 10 ng/μL. Normalised libraries were quantified again and equimolar and/or weighted 2.8 nM pools were created. Pool concentrations were checked using the Agilent 4200 TapeStation (Agilent) with High Sensitivity D500 reagents before sequencing. Sequencing was performed using paired-end 150 bp reads on the Illumina NovaSeq 6000.

### Genome assembly

Prior to assembly of the PacBio HiFi reads, a database of *k*-mer counts ( *k* = 31) was generated from the filtered reads using FastK. GenomeScope2 ( Ranallo-Benavidez *et al.*, 2020) was used to analyse the *k*-mer frequency distributions, providing estimates of genome size, heterozygosity, and repeat content.

The HiFi reads were assembled using Hifiasm in Hi-C phasing mode ( Cheng *et al.*, 2021; Cheng *et al.*, 2022), producing two haplotypes. Hi-C reads ( Rao *et al.*, 2014) were mapped to the primary contigs using bwa-mem2 ( Vasimuddin *et al.*, 2019). Contigs were further scaffolded with Hi-C data in YaHS ( Zhou *et al.*, 2023), using the --break option for handling potential misassemblies. The scaffolded assemblies were evaluated using Gfastats ( Formenti *et al.*, 2022), BUSCO ( Manni *et al.*, 2021) and MERQURY.FK ( Rhie *et al.*, 2020). The organelle genomes were assembled using MitoHiFi ( Uliano-Silva *et al.*, 2023).

### Assembly curation

The assembly was decontaminated using the Assembly Screen for Cobionts and Contaminants ( ASCC) pipeline. TreeVal was used to generate the flat files and maps for use in curation. Manual curation was conducted primarily in PretextView and HiGlass ( Kerpedjiev *et al.*, 2018). Scaffolds were visually inspected and corrected as described by Howe *et al*. (2021). Manual corrections included 63 breaks and 167 joins, which reduced the scaffold count by 3.3%. The curation process is described at https://gitlab.com/wtsi-grit/rapid-curation. PretextSnapshot was used to generate a Hi-C contact map of the final assembly.

### Assembly quality assessment

The Merqury.FK tool ( Rhie *et al.*, 2020) was run in a Singularity container ( Kurtzer *et al.*, 2017) to evaluate *k*-mer completeness and assembly quality for both haplotypes using the *k*-mer databases ( *k* = 31) computed prior to genome assembly. The analysis outputs included assembly QV scores and completeness statistics.

The genome was analysed using the BlobToolKit pipeline, a Nextflow implementation of the earlier Snakemake version ( Challis *et al.*, 2020). The pipeline aligns PacBio reads using minimap2 ( Li, 2018) and SAMtools ( Danecek *et al.*, 2021) to generate coverage tracks. It runs BUSCO ( Manni *et al.*, 2021) using lineages identified from the NCBI Taxonomy ( Schoch *et al.*, 2020). For the three domain-level lineages, BUSCO genes are aligned to the UniProt Reference Proteomes database ( Bateman *et al.*, 2023) using DIAMOND blastp ( Buchfink *et al.*, 2021). The genome is divided into chunks based on the density of BUSCO genes from the closest taxonomic lineage, and each chunk is aligned to the UniProt Reference Proteomes database with DIAMOND blastx. Sequences without hits are chunked using seqtk and aligned to the NT database with blastn ( Altschul *et al.*, 1990). The BlobToolKit suite consolidates all outputs into a blobdir for visualisation. The BlobToolKit pipeline was developed using nf-core tooling ( Ewels *et al.*, 2020) and MultiQC ( Ewels *et al.*, 2016), with containerisation through Docker ( Merkel, 2014) and Singularity ( Kurtzer *et al.*, 2017).

## Genome sequence report

### Sequence data

**Figure 2.**
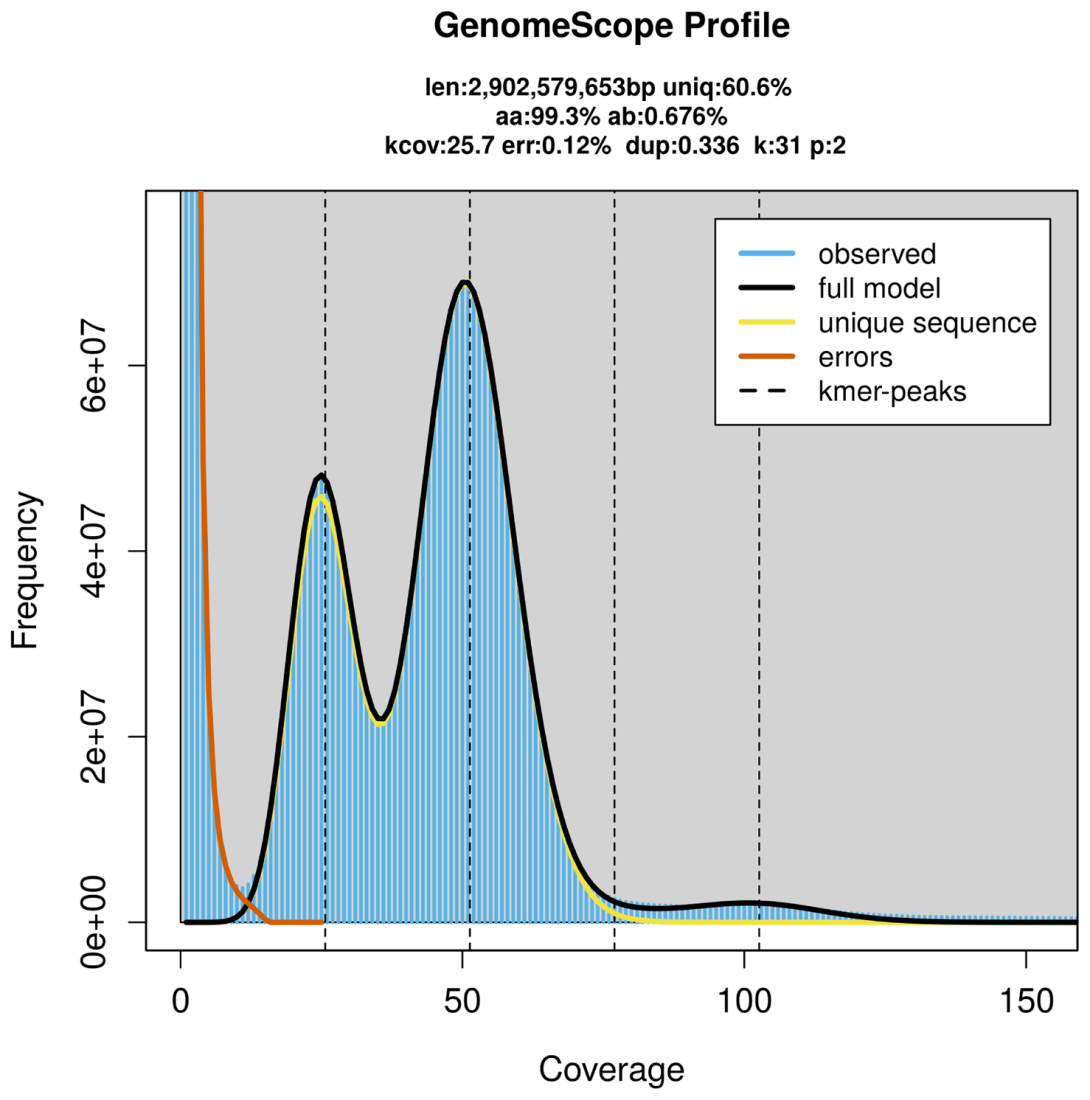
Frequency distribution of *k*-mers generated using GenomeScope2. The plot shows observed and modelled *k*-mer spectra, providing estimates of genome size, heterozygosity, and repeat content based on unassembled sequencing reads.

PacBio sequencing of the *Araneus angulatus* specimen generated 129.23 Gb (gigabases) from 12.35 million reads, which were used to assemble the genome. GenomeScope2.0 analysis estimated the haploid genome size at 2 902.58 Mb, with a heterozygosity of 0.68% and repeat content of 39.48% ( Figure 2). These estimates guided expectations for the assembly. Based on the estimated genome size, the sequencing data provided approximately 51× coverage. Hi-C sequencing produced 239.79 Gb from 1 588.02 million reads, which were used to scaffold the assembly. Table 1 summarises the specimen and sequencing details.

**Table 1.** Specimen and sequencing data for BioProject PRJEB73665.

Platform	PacBio HiFi	Hi-C
**ToLID**	qqAraAngu1	qqAraAngu1
**Specimen ID**	NHMUK014537398	NHMUK014537398
**BioSample (source individual)**	SAMEA11024980	SAMEA11024980
**BioSample (tissue)**	SAMEA11025175	SAMEA11025178
**Tissue**	abdomen	cephalothorax
**Instrument**	Sequel IIe	Illumina NovaSeq 6000
**Run accessions**	ERR12736889; ERR12736890; ERR12736891; ERR12736892	ERR12737284
**Read count total**	12.35 million	1 588.02 million
**Base count total**	129.23 Gb	239.79 Gb

### Assembly statistics

The genome was assembled into two haplotypes using Hi-C phasing. Haplotype 1 was curated to chromosome level, while haplotype 2 was assembled to scaffold level. The final assembly has a total length of 2 980.91 Mb in 1 908 scaffolds, with 499 gaps, and a scaffold N50 of 102.38 Mb ( Table 2).

**Table 2.** Genome assembly statistics.

**Assembly name**	qqAraAngu1.hap1.1	qqAraAngu1.hap2.1
**Assembly accession**	GCA_965645275.1	GCA_965645295.1
**Assembly level**	chromosome	scaffold
**Span (Mb)**	2 980.91	2 941.08
**Number of chromosomes**	28	scaffold-level
**Number of contigs**	2 407	1 259
**Contig N50**	10.18 Mb	11.16 Mb
**Number of scaffolds**	1 908	779
**Scaffold N50**	102.38 Mb	97.17 Mb
**Longest scaffold length (Mb)**	160.13	
**Organelles**	Mitochondrion: 14.53 kb	

**Figure 3.**
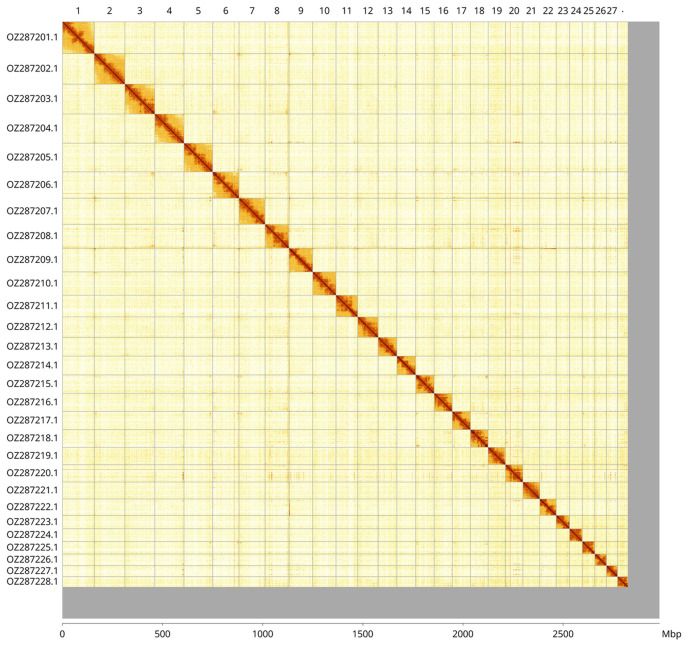
Hi-C contact map of the *Araneus angulatus* genome assembly. Assembled chromosomes are shown in order of size and labelled along the axes, with a megabase scale shown below. The plot was generated using PretextSnapshot.

Most of the haplotype 1 assembly sequence (94.74%) was assigned to 28 chromosomal-level scaffolds. These chromosome-level scaffolds, confirmed by Hi-C data, are named according to size ( Figure 3; Table 3). Sex chromosomes could not be identified, due to the lack of a related comparator. Scaffolds on Chromosome 18 in the regions 9.7–16.1 Mb and 76.5-85.3 Mb have uncertain order and orientation.

**Table 3.** Chromosomal pseudomolecules in the haplotype 1 genome assembly of *Araneus angulatus* qqAraAngu1.

INSDC accession	Molecule	Length (Mb)	GC%
OZ287201.1	1	160.13	33
OZ287202.1	2	152.26	33.50
OZ287203.1	3	149.62	33.50
OZ287204.1	4	145.17	33
OZ287205.1	5	143.20	32.50
OZ287206.1	6	132.08	33
OZ287207.1	7	129.73	33
OZ287208.1	8	119.32	33
OZ287209.1	9	118.51	33
OZ287210.1	10	116.54	33
OZ287211.1	11	107.85	33
OZ287212.1	12	102.38	33.50
OZ287213.1	13	94.09	33
OZ287214.1	14	93.29	32.50
OZ287215.1	15	92.47	33
OZ287216.1	16	90.60	33.50
OZ287217.1	17	90.35	33.50
OZ287218.1	18	87.93	34
OZ287219.1	19	87.13	34
OZ287220.1	20	86.29	34
OZ287221.1	21	84.42	33.50
OZ287222.1	22	82.67	32.50
OZ287223.1	23	65.91	34
OZ287224.1	24	64.04	33
OZ287225.1	25	62.51	33.50
OZ287226.1	26	57.99	33.50
OZ287227.1	27	54.69	33
OZ287228.1	28	53	34

The mitochondrial genome was also assembled (length 14.53 kb, OZ287229.1). This sequence is included as a contig in the multifasta file of the genome submission and as a standalone record.

**Figure 4.**
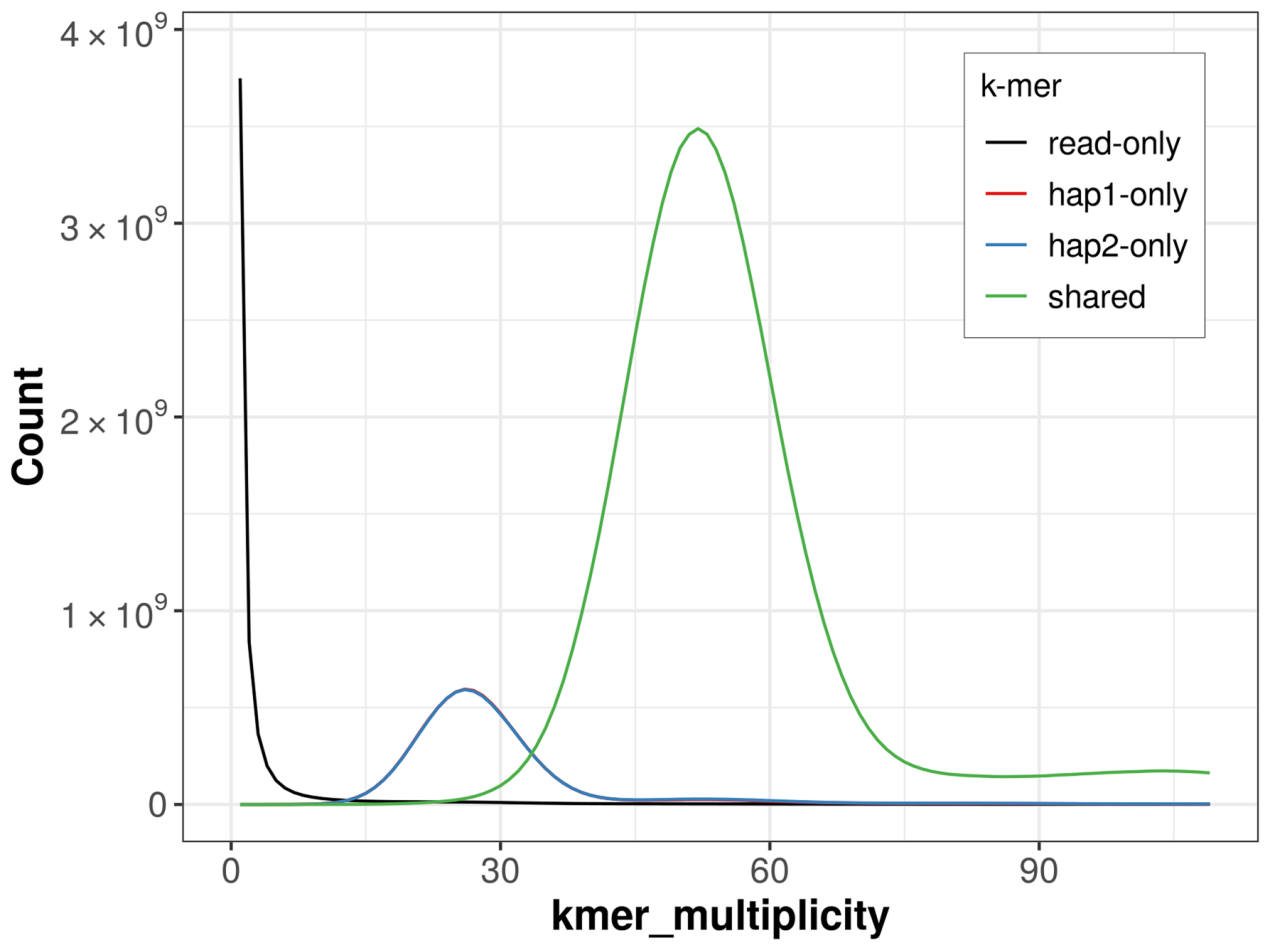
Evaluation of *k*-mer completeness using MerquryFK. This plot illustrates the recovery of *k*-mers from the original read data in the final assemblies. The horizontal axis represents *k*-mer multiplicity, and the vertical axis shows the number of *k*-mers. The black curve represents *k*-mers that appear in the reads but are not assembled. The green curve corresponds to *k*-mers shared by both haplotypes, and the red and blue curves show *k*-mers found only in one of the haplotypes.

For haplotype 1, the estimated QV is 59.2, and for haplotype 2, 60.2. When the two haplotypes are combined, the assembly achieves an estimated QV of 59.7. The *k*-mer completeness is 84.41% for haplotype 1, 84.43% for haplotype 2, and 99.13% for the combined haplotypes ( Figure 4).

**Figure 5.**
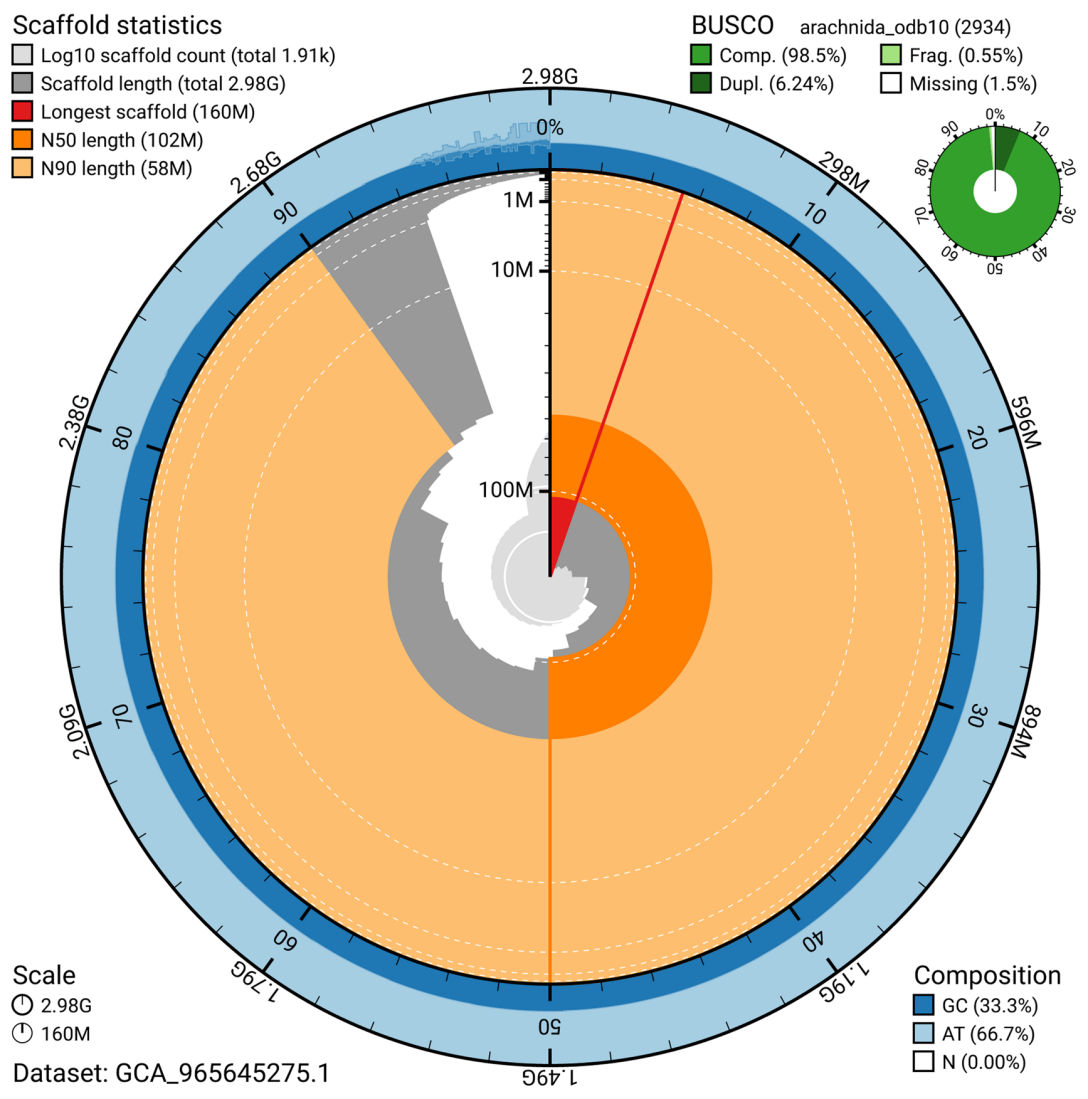
Assembly metrics for qqAraAngu1.hap1.1. The BlobToolKit snail plot provides an overview of assembly metrics and BUSCO gene completeness. The circumference represents the length of the whole genome sequence, and the main plot is divided into 1 000 bins around the circumference. The outermost blue tracks display the distribution of GC, AT, and N percentages across the bins. Scaffolds are arranged clockwise from longest to shortest and are depicted in dark grey. The longest scaffold is indicated by the red arc, and the deeper orange and pale orange arcs represent the N50 and N90 lengths. A light grey spiral at the centre shows the cumulative scaffold count on a logarithmic scale. A summary of complete, fragmented, duplicated, and missing BUSCO genes in the set is presented at the top right. An interactive version of this figure can be accessed on the BlobToolKit viewer.

**Figure 6.**
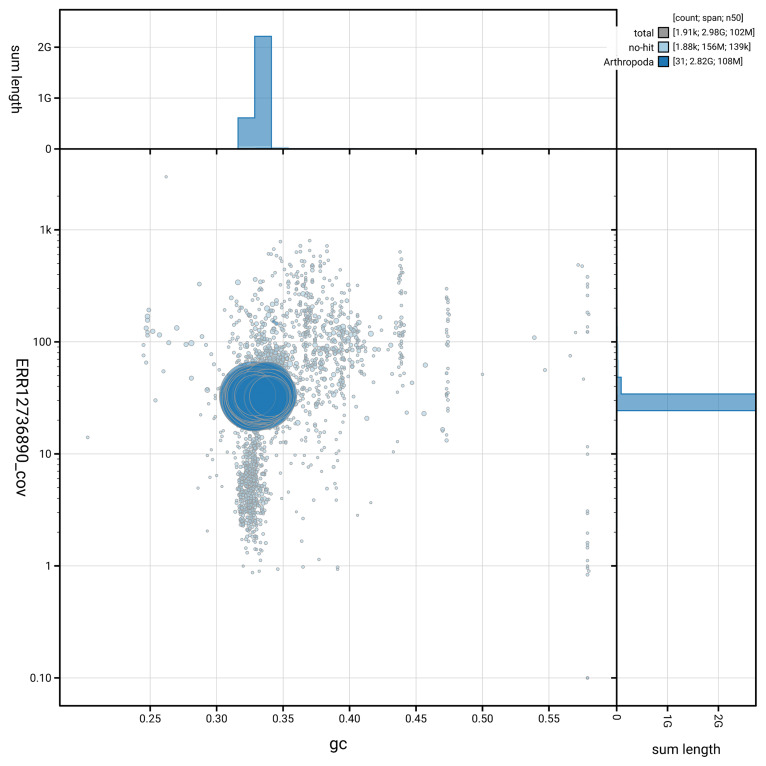
BlobToolKit GC-coverage plot for qqAraAngu1.hap1.1. Blob plot showing sequence coverage (vertical axis) and GC content (horizontal axis). The circles represent scaffolds, with the size proportional to scaffold length and the colour representing phylum membership. The histograms along the axes display the total length of sequences distributed across different levels of coverage and GC content. An interactive version of this figure is available on the BlobToolKit viewer.

BUSCO analysis using the arachnida_odb10 reference set ( *n* = 2 934) identified 98.5% of the expected gene set (single = 92.3%, duplicated = 6.2%) for haplotype 1. The snail plot in Figure 5 summarises the scaffold length distribution and other assembly statistics for haplotype 1. The blob plot in Figure 6 shows the distribution of scaffolds by GC proportion and coverage for haplotype 1.

Table 4 lists the assembly metric benchmarks adapted from Rhie *et al.* (2021) and the Earth BioGenome Project Report on Assembly Standards September 2024. The EBP metric, calculated for the haplotype 1, is **7.C.Q59**, meeting the recommended reference standard.

**Table 4.** Earth Biogenome Project summary metrics for the *Araneus angulatus* assembly.

Measure	Value	Benchmark
EBP summary (haplotype 1)	7.C.Q59	6.C.Q40
Contig N50 length	10.18 Mb	≥ 1 Mb
Scaffold N50 length	102.38 Mb	= chromosome N50
Consensus quality (QV)	Haplotype 1: 59.2; haplotype 2: 60.2; combined: 59.7	≥ 40
*k*-mer completeness	Haplotype 1: 84.41%; Haplotype 2: 84.43%; combined: 99.13%	≥ 95%
BUSCO	C:98.5% [S:92.3%; D:6.2%]; F:0.5%; M:1.0%; n:2 934	S > 90%; D < 5%
Percentage of assembly assigned to chromosomes	94.74%	≥ 90%

### Wellcome Sanger Institute – Legal and Governance

The materials that have contributed to this genome note have been supplied by a Darwin Tree of Life Partner. The submission of materials by a Darwin Tree of Life Partner is subject to the **‘Darwin Tree of Life Project Sampling Code of Practice’**, which can be found in full on the Darwin Tree of Life website. By agreeing with and signing up to the Sampling Code of Practice, the Darwin Tree of Life Partner agrees they will meet the legal and ethical requirements and standards set out within this document in respect of all samples acquired for, and supplied to, the Darwin Tree of Life Project. Further, the Wellcome Sanger Institute employs a process whereby due diligence is carried out proportionate to the nature of the materials themselves, and the circumstances under which they have been/are to be collected and provided for use. The purpose of this is to address and mitigate any potential legal and/or ethical implications of receipt and use of the materials as part of the research project, and to ensure that in doing so we align with best practice wherever possible. The overarching areas of consideration are:

- Ethical review of provenance and sourcing of the material
- Legality of collection, transfer and use (national and international)

Each transfer of samples is further undertaken according to a Research Collaboration Agreement or Material Transfer Agreement entered into by the Darwin Tree of Life Partner, Genome Research Limited (operating as the Wellcome Sanger Institute), and in some circumstances, other Darwin Tree of Life collaborators.

## Data Availability

European Nucleotide Archive: Araneus angulatus. Accession number PRJEB73665. The genome sequence is released openly for reuse. The *Araneus angulatus* genome sequencing initiative is part of the Darwin Tree of Life Project (PRJEB40665) and the Sanger Institute Tree of Life Programme (PRJEB43745). All raw sequence data and the assembly have been deposited in INSDC databases. The genome will be annotated using available RNA-Seq data and presented through the Ensembl pipeline at the European Bioinformatics Institute. Raw data and assembly accession identifiers are reported in Table 1 and Table 2. Production code used in genome assembly at the WSI Tree of Life is available at https://github.com/sanger-tol. Table 5 lists software versions used in this study.

## Author information

Contributors are listed at the following links:

- Members of the Natural History Museum Genome Acquisition Lab
- Members of the Darwin Tree of Life Barcoding collective
- Members of the Wellcome Sanger Institute Tree of Life Management, Samples and Laboratory team
- Members of Wellcome Sanger Institute Scientific Operations – Sequencing Operations
- Members of the Wellcome Sanger Institute Tree of Life Core Informatics team
- Members of the Tree of Life Core Informatics collective
- Members of the Darwin Tree of Life Consortium
