# Supplementary material for: The genome sequence of an orb-weaver spider, Araneus angulatus Clerck, 1757 (Araneae: Araneidae)
Source: Wellcome Open Res. 2026 Oct 20;10:656. Originally published 2025 Nov 21. [Version 2] doi: 10.12688/wellcomeopenres.25196.2 (PMC12780517; doi:10.12688/wellcomeopenres.25196.2)
Supplement: Peer review history

# Reviewer report: Reviewer response for version 1

**DOI:** 10.21956/wellcomeopenres.27772.r143716

**Published:** 2026-01-07

**Authors:** Bastian Fromm

**Affiliations:** 1 UiT- The Arctic University of Norway, Tromsø, Norway

Not sure if this is a fully automatically generated article, as I have received another review request for a spider genome for Wellcome Open Research that is written exactly the same way (and, hence, will get the same review). 

 The report is state-of-the-art report of genome assembly techniques and statistics of a spider ( *Araneus angulatus*).

 I am personally surprised by the lack of a genome annotation and would like to ask the authors for a statement why it was not included. 

 I like the relatively extensive introduction to the species.

Are sufficient details of methods and materials provided to allow replication by others?

Yes

Is the rationale for creating the dataset(s) clearly described?

Yes

Are the datasets clearly presented in a useable and accessible format?

Yes

Are the protocols appropriate and is the work technically sound?

Yes

Reviewer Expertise:

non-coding genes

I confirm that I have read this submission and believe that I have an appropriate level of expertise to confirm that it is of an acceptable scientific standard.

# Reviewer report: Reviewer response for version 1

**DOI:** 10.21956/wellcomeopenres.27772.r142355

**Published:** 2025-12-30

**Authors:** Adile Akpınar

**Affiliations:** 1 Gaziantep University, Gaziantep, Turkey

Morphological data for the species *Araneus angulatus* may be insufficient for species identification and may lead to synonmy.This article presents a comprehensive genomic study that details all molecular data for *Araneus angulatus.* The results of this article serve as a reference for future studies. There are many closely related species among spiders, which can lead to misidentification of species and synonymy.

Are sufficient details of methods and materials provided to allow replication by others?

Yes

Is the rationale for creating the dataset(s) clearly described?

Yes

Are the datasets clearly presented in a useable and accessible format?

Yes

Are the protocols appropriate and is the work technically sound?

Yes

Reviewer Expertise:

Araneae taxonomy

I confirm that I have read this submission and believe that I have an appropriate level of expertise to confirm that it is of an acceptable scientific standard.
